# Insecticide resistance profiles of *Anopheles gambiae* s.l. in Togo and genetic mechanisms involved, during 3-year survey: is there any need for resistance management?

**DOI:** 10.1186/s12936-019-2813-z

**Published:** 2019-05-22

**Authors:** Adjovi D. Amoudji, Koffi M. Ahadji-Dabla, Aristide Sawdetuo Hien, Yawo Georges Apétogbo, Bienvenu Yaméogo, Diloma Dieudonné Soma, Rabila Bamogo, Rachid Tinah Atcha-Oubou, Roch Kounbobr Dabiré, Guillaume Koffivi Ketoh

**Affiliations:** 1 Department of Zoology and Animal Biology, Faculty of Sciences, University of Lomé, 01 B.P. 1515 Lomé 01, Togo; 2 Biodiversity Institute & Department of Ecology and Evolutionary Biology, University of Kansas, Lawrence, KS 66045 USA; 3 Institut de Recherche en Sciences de la Santé/Centre Muraz, 01 BP 545, Bobo-Dioulasso 01, Burkina Faso; 4 National Malaria Control Program/Ministry of Health, 01 B.P. 518 Lomé 01, Togo

**Keywords:** Malaria, *Anopheles gambiae* s.l., Resistance, *kdr*, *ace*-*1*, Vector control, Togo

## Abstract

**Background:**

Malaria, one of the world’s greatest public health challenges, is an endemic disease with stable transmission in Togo. Combating malaria requires an effective vector control. This study provides temporal data on insecticide resistance status in the major malaria vector *Anopheles gambiae* sensu lato (s.l.) from Togo.

**Methods:**

Two to 5 days old females of *An. gambiae* s.l., originating from three localities (Baguida, Kovié, Kolokopé) were subjected to insecticide-impregnated papers during 3 years (2012, 2013, 2016) as follows: organochlorides (4% DDT), pyrethroids (0.05% deltamethrin, 0.75% permethrin, 0.05% lambdacyhalothrin), carbamates (0.4% bendiocarb and 0.1% propoxur), and organophosphates (5% malathion, 0.4% chlorpyrifos methyl, 1% fenitrothion) following the WHO standard protocol. Dead and surviving mosquitoes were stored separately in Eppendorf tubes containing silica gel for DNA extraction, species identification, and *kdr* and *ace*-*1* genotyping.

**Results:**

Knockdown times (KDT_50_ and KDT_95_) were high in *An. gambiae* s.l. The lowest KDTs were recorded at Baguida in 2013 for deltamethrin (KDT_50_ = 24.7, CI [22.4–27.12] and KDT_95_ = 90.78, CI [76.35–113.49]). No KDTs were recorded for DDT and in some instances for permethrin. In general, *An. gambiae* s.l. was resistant to most of the four classes of insecticides during the survey periods regardless of locality and year, except to chlorpyrifos methyl. In some instances, mosquitoes were fully susceptible to fenitrothion (Kolokopé: 100% and Kovié: 98.05%, CI [95.82–100.26]) and malathion (100% at both Kolokopé and Kovié) in 2013, and malathion only (Kolokopé; 100%) in 2016. *Anopheles coluzzii*, *An. gambiae* and *Anopheles arabiensis* were the three sibling species identified at the three localities with some hybrids at Baguida (2013), and Kovié (2012 and 2016), respectively. *Anopheles gambiae* was relatively dominant (61.6%). The *kdr* 1014F allele frequency was > 0.9 in most of the cases, except at Kolokopé (f (1014F) = 0.63, CI [0.55–0.71]) in 2013. The *kdr* 1014S allele frequency was below 0.02. The highest *ace*-*1* frequencies were identified in *An. gambiae* at Baguida (2012: 0.52, CI [0.34–0.69] and 2013: 0.66, CI [0.46–0.86]).

**Conclusion:**

The resistance status is worrying in Togo and should be considered in future malaria vector resistance management programmes by decision-makers.

**Electronic supplementary material:**

The online version of this article (10.1186/s12936-019-2813-z) contains supplementary material, which is available to authorized users.

## Background

Although the global response to malaria was considered one of the world’s greatest public health achievements, the overall decline in the global malaria burden has levelled off [1]. In 2017, there were an estimated 219 million cases of malaria worldwide, mostly occurring in the World Health Organization (WHO) African Region (92%) [2].

In Togo, malaria is endemic with a stable transmission [3]. The control of the disease is one of the Ministry of Health’s priorities [4]. Since 1998, Togo has adhered to the Roll Back Malaria (RBM) Initiative launched in October 1998, with the objective to reduce the malaria burden which represents an obstacle to development in endemic countries, by 2030. Malaria control in Togo focuses mainly on two strategies implemented by the National Malaria Control Programme (NMCP), including prevention and clinical management. Despite efforts to overcome the scourge, malaria remains a major public health concern. In 2016, it was reported by the NMCP that malaria morbidity was 38% of outpatient consultations and 17% of hospital admissions; children under 5 years of age were the most affected. Mortality in infants was 67.7% [5].

The primary tool for malaria vector control in Togo is long-lasting insecticide-treated bed nets (LLINs) [6]. Other means, including mosquito coils and aerosols, are also used by households [7]. However, insecticide resistance in malaria vector populations widespread in West Africa, could impede the success of malaria control programmes in general. In Togo, reports have indicated resistance of *Anopheles gambiae* sensu lato (s.l.), the major malaria vector, to the four classes of insecticides available for public health [5, 7–10].

Both enhanced detoxification [11] and mutations in the gene encoding the voltage-gated sodium channel knockdown resistance (*kdr*), and insensitive acetylcholinesterase (*ace*-*1*) [12] have been reported as the most important mechanisms involved in insecticide resistance. Two amino acid changes in the sodium channel gene at codon 1014 are involved in *kdr* in *An. gambiae* s.s.: a leucine to phenylalanine substitution (1014F) [13] and a leucine to serine substitution (1014S) [14]. Consequently, target site mutations (i.e., 1014F and 1014S) confer resistance to pyrethroids and DDT in *An. gambiae* sensu stricto (s.s.). Insensitive acetylcholinesterase gene confers resistance to both organophosphates and carbamates in *An. gambiae* s.s. through a single amino acid substitution of glycine to serine at position 119 [15].

Malaria vector resistance, which is widespread in Africa, particularly in Benin [16–18], Burkina Faso [19–21], Ghana [22, 23], Cameroon [24, 25], and Kenya [26], constitutes the first challenge to achieving a decline in malaria cases. Hence, knowledge of insecticide resistance is a basic requirement that will guide the use of insecticides in malaria control programmes. This study presented a 3-year survey of insecticide resistance profiles of *An. gambiae* s.l. in three agricultural localities of Togo. Dynamics of *An. gambiae* species and the frequency of *kdr* (L1014F and L1014S) and *ace*-*1* mutations were also investigated.

## Methods

### Study area

**Fig. 1:**
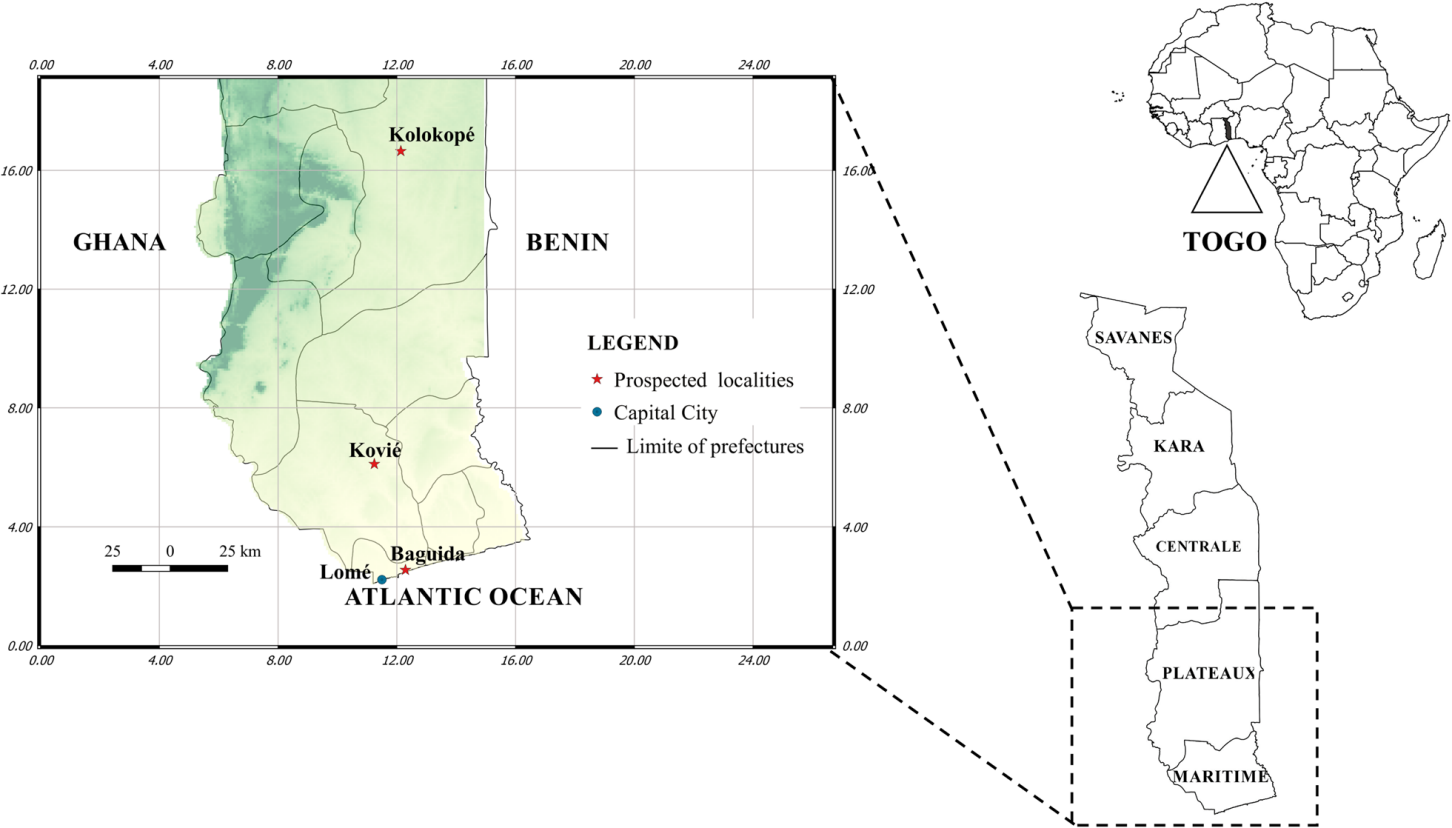
Map showing the collection sites of *Anopheles gambiae* s.l.

This study was conducted at three localities characterized by different agricultural ecosystems in Togo: Baguida, Kovié, and Kolokopé (see Fig. 1). Baguida (06°09′47″N–01°19′05″E) is a peri-urban area with vegetable production located at the coastal part of Togo. Kovié (06º34′38″N–01º11′47″E) is a rural area mainly characterized by rice cultivation. This locality is 30 km from Lomé. Baguida and Kovié are located at the southern part (Région Maritime) of the country. Kolokopé (06º34′38″N–01º11′47″E), a cotton-growing area is a rural locality 250 km from Lomé in the highlands (Région des Plateaux) of Togo.

The south of Togo is characterized by a sub-equatorial climate with two dry seasons (December to March and August to September) and two rainy seasons (April to July and October to November). The highlands are characterized by a microclimate commonly known as mountain climate.

### Mosquito collection

Different instar larvae of *An. gambiae* s.l. were collected over a period of 3 years (i.e., 2012, 2013, 2016) at Kolokopé, Kovié and Baguida. Larvae were sampled between June and July of each year during the rainy season from permanent and semi-permanent water collection points and pooled together by locality. Samples were transported to the insectary for rearing under standard conditions (25 ± 2 °C, 80 ± 4% [relative humidity]).

### Insecticide susceptibility assays

Two to 5 days old F_0_ female mosquitoes were used for insecticide susceptibility tests. Insecticide bioassays were conducted following the WHO protocol [27, 28]. The following insecticides were tested: organochlorides (4% DDT), pyrethroids (0.05% deltamethrin, 0.75% permethrin, 0.05% lambdacyhalothrin), carbamates (0.4% bendiocarb and 0.1% propoxur), and organophosphates (5% malathion, 0.4% chlorpyrifos methyl, 1% fenitrothion).

Twenty to 25 unfed females were randomly selected and exposed to each impregnated paper for 1 h, except for fenitrothion which exposure time was 2 h. The knockdown effect of pyrethroids and DDT were observed every 10 min for 1 h and individuals knocked down were recorded. A susceptible Kisumu strain of *An. gambiae* was used as a reference for the bioassays. Before their exposition, the mosquitoes were observed for 1 h in holding tubes. After exposure, the mosquitoes were transferred to the holding tubes and fed with 10% glucose solution [27, 28]. Dead individuals were recorded 24-h post-exposure. A total of 5888 F_0_ female mosquitoes were used for the bioassays: Baguida (n = 2067), Kovié (n = 1932) and Kolokopé (n = 1889) (see Table 1). Dead and surviving mosquitoes were stored separately in Eppendorf tubes containing silica gel and transported to *Institut de Recherche en Sciences de la Santé* (IRSS, Bobo-Dioulasso, Burkina Faso) for species identification and resistance mechanism characterization.

**Table 1:** Susceptibility status of *An. gambiae* s.l. collected in Togo and Kisumu exposed to the four classes of insecticide

Year	Insecticides	Strain	N	Mortality (%)	[95% CI]	Susceptibility status
2012	4% DDT	Baguida	103	10.68	[2.71–18.23]	Resistant
		Kolokopé	95	0	0	Resistant
		Kovié	98	1.02	[0.15–3.22]	Resistant
		Kisumu	103	100	100	Susceptible
	0.05% Deltamethrin	Baguida	99	32.32	[23.23–41.62]	Resistant
		Kolokopé	101	3.96	[0.77–6.93]	Resistant
		Kovié	95	33.68	[20.2–46.4]	Resistant
		Kisumu	98	100	100	Susceptible
	0.1% Bendiocarb	Baguida	96	7.29	[1.19–13.8]	Resistant
		Kolokopé	99	89.89	[82.32–97.42]	Resistant
		Kovié	94	60.63	[45.77–76.48]	Resistant
		Kisumu	106	100	100	Susceptible
	0.4% Chlorpyriphos methyl	Baguida	100	100	100	Susceptible
		Kolokopé	103	100	100	Susceptible
		Kovié	106	100	100	Susceptible
		Kisumu	97	100	100	Susceptible
2013	4% DDT	Baguida	97	6.18	[0.76–12.23]	Resistant
		Kolokopé	96	0	0	Resistant
		Kovié	90	15.55	[7.16–22.96]	Resistant
		Kisumu	99	100	100	Susceptible
	0.05% Deltamethrin	Baguida	87	52	[46.71–56.64]	Resistant
		Kolokopé	74	14.86	[7.79–21.92]	Resistant
		Kovié	90	22.22	[12.32–32.88]	Resistant
		Kisumu	101	100	100	Susceptible
	0.75% Permethrin	Baguida	89	20.22	[5.15–37.39]	Resistant
		Kolokopé	80	7.5	[0.98–14.3]	Resistant
		Kovié	90	26.26	[14.58–39.68]	Resistant
		Kisumu	98	100	100	Susceptible
	0.05% Lambdacyhalothrin	Baguida	90	57.77	[51.15–64.69]	Resistant
		Kolokopé	85	0	0	Resistant
		Kovié	81	14.81	[1.65–28.21]	Resistant
		Kisumu	108	100	100	Susceptible
	0.1% Bendiocarb	Baguida	87	9.19	[0.58–18.33]	Resistant
		Kolokopé	88	98.86	[96.92–101]	Susceptible
		Kovié	93	68.81	[59.84–77.1]	Resistant
		Kisumu	102	100	100	Susceptible
	0.1% Propoxur	Baguida	95	18.94	[10.3–26.95]	Resistant
		Kolokopé	97	97.93	[95.65–100.27]	Suspected resistant
		Kovié	89	73.03	[63.28–82.42]	Resistant
		Kisumu	98	100	100	Susceptible
	1% Fenitrothion	Baguida	94	20.21	[5.64–34.69]	Resistant
		Kolokopé	100	100	100	Susceptible
		Kovié	103	98.05	[95.82–100.26]	Susceptible
		Kisumu	96	100	100	Susceptible
2013	5% Malathion	Baguida	99	73.73	[59.79–88.33]	Resistant
		Kolokopé	90	100	100	Susceptible
		Kovié	98	100	100	Susceptible
		Kisumu	95	100	100	Susceptible
	0.4% Chlorpyrisphos methyl	Baguida	90	100	100	Susceptible
		Kolokopé	95	100	100	Susceptible
		Kovié	89	100	100	Susceptible
		Kisumu	102	100	100	Susceptible
2016	4% DDT	Baguida	121	1.61	[0.25–3.56]	Resistant
		Kolokopé	69	0	0	Resistant
		Kovié	98	0	0	Resistant
		Kisumu	101	100	100	Susceptible
	0.05% Deltamethrin	Baguida	123	17.03	[10.39–23.21]	Resistant
		Kolokopé	67	8.97	[0.66–16.01]	Resistant
		Kovié	91	4.4	[1.53–8.27]	Resistant
		Kisumu	92	100	100	Susceptible
	0.75% Permethrin	Baguida	72	0	0	Resistant
		Kolokopé	93	10.75	[5.13–25.69]	Resistant
		Kovié	73	6.06	[1.81–14.58]	Resistant
		Kisumu	96	98.96	[96.48–101.14]	Susceptible
	0.05% Lambdacyhalothrin	Baguida	121	16.53	[4.7–26.24]	Resistant
		Kolokopé	97	16.49	[7.22–26.04]	Resistant
		Kovié	88	2.27	[1.66–5.1]	Resistant
		Kisumu	90	98.89	[97.15–100.92]	Susceptible
	0.1% Bendiocarb	Baguida	111	2.7	[0.95–4.55]	Resistant
		Kolokopé	82	68.29	[55.5–80.42]	Resistant
		Kovié	81	1.23	[0.92–2.85]	Resistant
		Kisumu	87	98.85	[95.65–101.41]	Susceptible
	0.1% Propoxur	Baguida	75	6.67	[1.37–11.46]	Resistant
		Kolokopé	89	71.91	[64.86–80.37]	Resistant
		Kovié	99	8.08	[4.58–10.67]	Resistant
		Kisumu	83	98.80	[95.38–101.5]	Susceptible
	1% Fenitrothion	Baguida	101	47.52	[25.39–69.81]	Resistant
		Kolokopé	89	100	100	Resistant
		Kovié	87	82.76	[77.56–87.89]	Résistant
		Kisumu	95	100	100	Susceptible
	5% Malathion	Baguida	117	30.77	[28.18–33.08]	Resistant
		Kolokopé	89	100	100	Susceptible
		Kovié	99	92.93	[84.66–98.9]	Suspected resistant
		Kisumu	83	98.80	[96.48–101.14]	Susceptible

N, number of mosquitoes; CI, confidence interval

### *Anopheles gambiae* s.l. species identification

Each mosquito’s DNA was extracted from head and thorax with 2% cetyl trimethyl ammonium bromide (2% CTAB). *Anopheles gambiae* s.l. was identified to species by PCR according to the method of Santolamazza et al. [29].

Species identification was performed by PCR-SINE200 technique. The extracted DNA was amplified in 20 µl of a master mixture. Two specific primers were used: (5′-TCG-CCT TAG ACC TTG CGT TA-3′), (5′-CGC TTC AAG AAT TCG AGA TAC-3′). The amplification conditions were as follows: an initial step of activation of the DNA polymerase for 10 min at 94 °C followed by 35 cycles at 94 °C for 30 s, a hybridization at 54 °C for 30 s and at 72 °C for 1 min and a final elongation at 72 °C for 10 min followed by a decrease in temperature to 4 °C. The amplified products were analysed by electrophoresis on 2% agarose gel and stained with ethidium bromide and visualized under UV light. The following band sizes distinguish the species of the *An. gambiae* complex: 479 bp fragment for *An. coluzzii*, 249 bp for *An. gambiae* and 223 bp for *An. arabiensis*.

### *Kdr* and *ace*-*1* molecular genotyping

The presence of *kdr* 1014F and 1014S alleles also known as kdr-west (kdr-w) and kdr-east (kdr-e), respectively, were detected, respectively, by the methods of Martinez-Torres et al. [13] and Ranson et al. [14]. Presence of G119S ace-1 mutation was also investigated using the method of Weill et al. [15].

The West African kdr L1014F mutation was detected using the common primers Agd1 (5′-ATA GAT TCC CCG ACC ATG-3′) and Agd2 (5′-AGA CAA GGA TGA TGA ACC-3′), and Agd3 (5′-AAT TTG CAT TAC TTA CGA CA-3′) the susceptible primer and Agd4 (5′-CTG TAG TGA TAG GAA ATT TA-3′) the resistance primer. Amplification was performed under the following temperature conditions: DNA polymerase activation step at 94 °C for 3 s followed by 35 cycles at 94 °C for 30 s, a hybridization phase of 30 s at 55 °C and 10 s at 72 °C with final elongation for 5 min at 72 °C. The expected band sizes to distinguish resistant and susceptible in sibling species are: 293 bp fragment for the common band, 195 bp for resistant allele and 137 bp for susceptible allele.

Detection of the East African *kdr* allele, L1014S followed the same procedure as described above. The following primers were used for amplification: Agd1 (5′-ATA GAT TCC CCG ACC ATG-3′), Agd2 (5′-AGA CAA GGA TGA TGA ACC-3′), Agd4 (5′-CTG TAG TGA TAG GAA ATT TA-3′), and Agd5 (5′-ATT TGC ATT ACT TAC GAC TG-3′). Agd1, Agd2 were the common primers, Agd4 was the susceptible primer, and Agd5 the resistant primer.

The ace-1 G119S mutation was performed with the primers Ex3AGdir (5′-GAT CGT GGA CAC CGT GTT CG-3′), Ex3AGrev 5′-AGG ATG GCC CGC TGG AAC AG-3′ which were amplified in the following conditions: DNA polymerase activation step at 94 °C for 3 s followed by 35 cycles at 94 °C for 30 s, a hybridization phase for 30 s at 62 °C and 30 s at 72 °C with a final elongation at 72 °C for 5 min. After amplification, the products (5 µl) were analysed by electrophoresis on 2% agarose gel to verify the appearance of the expected first bands (541 bp). Fifteen microlitre of PCR product was digested with 5U of *Alu*I restriction enzyme in a final volume of 25 μl at 37 °C overnight.

Final products resulting from digestion were also analysed by electrophoresis to differentiate a 403 bp fragment for susceptible homozygous mosquitoes (SS), and two fragments of 253 bp and 150 bp for homozygous resistant (RR).

Samples from the localities accounting for the 3 years were analysed for the presence of *kdr*-*w* and *ace*-*1* (except those from Kolokopé 2013). Regarding *kdr*-*e*, only samples from Baguida (2012 and 2013), Kovié (2013) and Kolokopé (2012) were analysed.

### Data analysis

Knockdown times (KDT_50_ and KDT_95_) along with 95% confidence intervals (CI) were calculated using Polo Plus version 1.0 software. Mortality rates were calculated as the ratio between dead individuals and the total number of mosquitoes tested; 95% CI of the mortality rates were calculated using Excel software (Microsoft Corp). The resistance status was determined using the WHO criteria [28]. The dynamics of the kdr 1014F frequency of each sibling species were compared between years using Fisher Exact test. The genotype frequencies of *kdr* and *ace*-*1* loci in sibling species were compared to Hardy–Weinberg expectations using Fisher exact test implemented in GenePop (version 4) software.

## Results

### Knockdown times and mortality rates of *Anopheles gambiae* populations

No knockdown times (TKD_50_ and TKD_95_) were observed for DDT in none of the localities during the 3 years. For deltamethrin, the TKD_50_ and TKD_95_ were all high. They were recorded in individuals from Baguida regardless the year. They were 42.56 (CI [38.18–48.28]) and 131.73 (CI [101.36–198.77]) in 2012, 24.71 (CI [22.4–27.12]) and 90.78 (CI [76.35–113.49]) in 2013, and 56.92 (CI [51.55–64.68]) and 203.99 (CI [154.86–304.06]), respectively. The KDTs of permethrin were also high and obtained in only Baguida and Kovié in 2016 (see Additional file 1: Table S1).

Mortality rates of *An. gambiae* s.l. are shown in Table 1. Mosquitoes were resistant to DDT in all the localities with the highest mortality (15.55%, CI [7.16–22.96]) at Kovié; no mortality was recorded at Kolokopé (2012, 2013, 2016) and Kovié (2016). Resistance to pyrethroids was also observed in the three localities. The highest mortalities were recorded in 2013: deltamethrin (52%, CI [46.71–56.64]) and lambdacyhalothrin (57.57%, CI [51.15–64.69]) at Baguida, and permethrin (26.26%, CI [14.5–39.68]) at Kovié. Regarding carbamates, mosquitoes were only susceptible to bendiocarb (98.86%, CI [96.9–101]) and suspected resistant to propoxur (97.93%, CI [95.6–100.27]), both in 2013. Concerning organophosphates, mosquitoes were fully susceptible to chlorpyrifos methyl in all the localities during the 3-year survey, fully susceptible at Kolokopé (2013 and 2016) and suspected resistant (92.93%, CI [84.66–98.9]) in 2016 at Kovié, both to malathion. Susceptibility to fenitrothion was observed at Kolokopé and Kovié in 2013, and at Kovié (98.05%, CI [95.82–100.26]) in 2016. Kisumu, the laboratory strain was susceptible to all insecticides.

### Sibling species of *Anopheles gambiae* complex identified

Species identification was carried in 914 individuals of *An. gambiae* complex (see Table 2). Overall, *An. gambiae* was the most common species (61.6%) followed by *An. coluzzii* (35.45%). *Anopheles arabiensis* was less common (0.87%). Hybrids (i.e., *An. coluzzii*/*An. gambiae*) were also identified (2.51%).

**Table 2:** Distribution of the sibling species of *An. gambiae* s.l. populations from Togo

Study sites	Geographic references	Social environment	Climatic areas	Agricultural practices	*An. gambiae* s.l.	*An. gambiae*		*An. coluzzii*		*Hybrids*		*An. arabiensis*	
					N	N	%	N	%	N	%	N	%
Baguida 2012	06°09′47″N–01°19′05″E	Peri-urban	Sub-equatorial	Vegetables	177	176	99.44	0	–			1	0.56
Baguida 2013					112	100	89.29	8	7.14	1	0.89	3	2.68
Baguida 2016					18	9	50.00	9	50.00		–		–
Kolokopé 2012	07°47′59″N-01°18′00″E	Rural	Sub-equatorial	Cotton	81	80	98.77	0	–		–	1	1.23
Kolokopé 2013					274	138	50.36	136	49.64		–		–
Kolokopé 2016					64	47	73.44	15	23.44		–	2	3.13
Kovié 2012	(06º34′38″N-01º11′47″E	Rural	Sub-equatorial	Rice, vegetables	29	2	6.90	11	37.93	16	57.17	0	–
Kovié 2013					90	7	7.78	83	92.22		–		–
Kovié 2016					69	0	–	62	89.86	6	8.70	1	1.45
Total					914	559	(61.61%)	324	(35.45%)	23	(2.51%)	8	(0.87%)

In 2012, *An. gambiae* was more common at Baguida (99.44%) and Kolokopé (98.77%); *An. arabiensis* was less common (0.56% at Baguida and 1.23% at Kolokopé). No *An. coluzzii* was identified at both localities. At Kovié, the most common species was the hybrid (57.17%); *An. coluzzii* and *An. gambiae* were 37.93 and 6.9%, respectively.

In 2013, *An. gambiae* was dominant in Baguida (89.29%) and Kolokopé (50.36%); *An. arabiensis* was 2.68% at Baguida and absent at Kolokopé. At Kovié, *An. gambiae* was 7.78% whereas *An. coluzzii* dominated (92.22%); *An. arabiensis* was absent (Table 3).

**Table 3:** Distribution and frequency of the L1014F knockdown resistance (*kdr*) alleles of *An. gambiae* s.l. populations from Togo

Species	Agricultural practices	Sites	N	Genotypes			f (L1014F)	[95% CI]	p(HW)
				1014L	1014L	1014F			
				1014L	1014F	1014F			
*An. arabiensis*	Cotton	KOL 2012	1	0	0	1	0.98	[0.71–1.25]	–
		KOL 2016	3	0	0	3	0.98	[0.82–1.14]	–
	Vegetables	BAG 2012	1	0	0	1	0.98	[0.71–1.25]	–
		BAG 2013	2	0	0	2	0.98	[0.79–1.17]	–
*An. coluzzii*	Rice	KOV 2013	26	1	2	23	0.923	[0.82–1.03]	0.1167
		KOV 2016	61	0	1	60	0.992	[0.97–1.01]	–
	Cotton	KOL 2013	133	49	1	83	0.628	[0.55–0.71]	0.0000
		KOL 2016	15	1	0	14	0.933	[0.80–1.06]	–
	Vegetables	BAG 2013	2	0	0	2	0.98	[0.79–1.17]	–
		BAG 2016	9	0	0	9	0.98	[0.89–1.07]	–
*An. gambiae*	Cotton	KOL 2012	60	0	0	60	0.98	[0.94–1.02]	–
		KOL 2013	137	5	1	131	0.96	[0.93–0.99]	0.0000
		KOL 2016	30	0	0	30	0.98	[0.93–1.03]	–
	Vegetables	BAG 2012	142	0	0	142	0.98	[0.96–1.00]	–
		BAG 2013	62	0	0	62	0.98	[0.95–1.01]	–
		BAG 2016	14	0	0	14	0.98	[0.86–1.10]	–

KOV, Kovié; KOL, Kolokopé; BAG, Baguida; N, number of mosquitoes; f (L1014F), frequency of the L1014F resistant allele; CI, confidence interval; p(HW), probability of the exact test for goodness of fit Hardy–Weinberg equilibrium

In 2016, the proportion of *An. gambiae* was higher at Kolokopé (73.44%) than at Baguida (50%). *Anopheles coluzzii* was 50% at Baguida as well. *Anopheles arabiensis* was less common at Kolokopé and Kovié (3.13 and 1.45%, respectively).

During the 3 years, hybrids were exclusively identified at Baguida (2013), Kovié (2012) and Kolokopé (2016) in proportions of 0.89; 57.17 and 8.70%, respectively.

### Genotype of target site insensitivity mutations

The *kdr* 1014F (kdr-w) mutation frequency is shown in Table 3. In any case, the kdr 1014F frequency was > 0.9 in *An. gambiae* and *An. arabiensis* regardless of year and locality. It was 0.98 at Kolokopé and Baguida in both species and 0.96 in *An. gambiae* at Kolokopé in 2013. In *An. coluzzii*, the frequency was also > 0.9 at all localities and in all years except at Kolokopé where it was 0.68 (CI [0.55–0.71]) in 2013. When kdr-w frequencies of all the localities were pooled together and sorted by year, the temporal distribution showed an increase of the frequency from 0.68 to 0.98 in *An. coluzzii* (see Table 4). This increase was especially observed at Kolokopé, which is a cotton-growing area (Table 3).

**Table 4:** Temporal distribution of 1014F kdr frequency in *An. gambiae* s.l. populations from Togo

Species	Year	N	Genotypes			f (L1014F)	[95% CI]
			1014L	1014L	1014F		
			1014L	1014F	1014F		
*An. arabiensis*	2012	2	0	0	2	0.98	[0.79–1.17]
	2013	2	0	0	2	0.98	[0.79–1.17]
	2016	3	0	0	3	0.98	[0.82–1.14]
*An. coluzzii*	2013	161	50	3	108	0.680	[0.61–0.75]
	2016	85	1	1	83	0.982	[0.95–1.01]
*An. gambiae*	2012	202	0	0	202	0.98	[0.96–1.00]
	2013	199	5	1	193	0.972	[0.95–0.99]
	2016	35	0	0	35	0.98	[0.93–1.03]

N, number of mosquitoes; f (L1014F), frequency of the L1014F allele; CI, confidence interval

The frequency of *kdr* 1014S (kdr-e) was 0.043 in *An. gambiae* from Kolokopé (2012) (Table 5). This mutation was not detected in samples from other localities.

**Table 5:** Frequency of the L1014S knock down resistance (*kdr*) alleles of *An. gambiae* s.l. populations from Togo

Species	Agricultural practices	Sites	N	Genotypes			f (L1014S)	[95% CI]	p(HW)
				1014L	1014L	1014S			
				1014L	1014S	1014S			
*An. arabiensis*	Vegetables	BAG 2012	1	1	0	0	0.000	–	–
*An. coluzzii*	Rice	KOV 2013	13	13	0	0	0.000	–	–
*An. gambiae*	Rice	KOV 2013	3	3	0	0	0.000	–	–
	Cotton	KOL 2012	23	22	0	1	0.043	[0.00–0.13]	0.024
	Vegetables	BAG 2012	36	36	0	0	0.000	–	–
		BAG 2013	16	16	0	0	0.000	–	–

KOV, Kovié; KOL, Kolokopé; BAG, Baguida; N, number of mosquitoes; f (L1014S), frequency of the L1014S resistant allele; CI, confidence interval; p(HW), probability of the exact test for goodness of fit Hardy–Weinberg equilibrium

The frequency of *ace 1* 119S was low in *An. coluzzii* at Kovié in 2013 (0.14, CI [0.03–0.24] and 2016 (0.05, CI [0.00–0.19]) and in hybrids (0.063, CI [0.00–0.18]) in 2012, and relatively higher in *An. gambiae* at Baguida in 2012 (0.52, CI [0.34–0.69]) and 2013 (0.66, CI [0.46–0.86]) (Table 6). No *An. arabiensis* in the samples was found with *ace*-*1* allele.

**Table 6:** Allelic and genotypic frequencies at the *ace*-*1* locus in *An. gambiae* s.l. populations from Togo

Species	Agricultural practices	Sites	N	Genotypes			f (119S)	[95% CI]	p(HW)
				119G	119G	119S			
				119G	119S	119S			
*An. coluzzii*	Rice	KOV 2012	11	11	0	0	0.000	–	–
		KOV 2013	40	29	11	0	0.138	[0.03–0.24]	1.0000
		KOV 2016	10	9	1	0	0.050	[0.00–0.19]	–
	Cotton	KOL 2016	9	9	0	0	0.000	–	–
	Vegetables	BAG 2016	1	1	0	0	0.000	–	–
*An. gambiae*	Rice	KOV 2012	2	2	0	0	0.000	–	–
	Cotton	KOL 2012	18	18	0	0	0.000	–	
		KOL 2016	8	8	0	0	0.000	–	–
	Vegetables	BAG 2012	31	6	18	7	0.516	[0.34–0.69]	0.4850
		BAG 2013	22	2	11	9	0.659	[0.46–0.86]	1.0000
*Hybrid*	Rice	KOV 2012	16	15	0	1	0.063	[0.00–0.18]	0.0341

KOV, Kovié; KOL, Kolokopé; BAG, Baguida; N, number of mosquitoes; f (119S), frequency of the 119S resistant allele; CI, confidence interval; p(HW), probability of the exact test for goodness of fit Hardy–Weinberg equilibrium

## Discussion

This study showed that levels of insecticide resistance in *Anopheles* species are high in Togo, given the high knockdown times, the low mortality rates recorded, and the high allele frequencies. Mosquitoes were especially resistant to DDT and pyrethroids, which target the voltage-gate sodium channel in *Anopheles* populations. Findings in this study outline the presence of three sibling species of the malaria vector *An. gambiae* s.l. In those species, *kdr* allele frequencies were high over the 3-year survey (*An. gambiae* and *An. arabiensis*) and increased significantly from 2013 to 2016 in *An. coluzzii*. This is the first time kdr 1014F mutation was observed in *An. arabiensis* at such a high frequency (i.e., f = 0.98). Knockdown resistance mutation was previously reported in *An. gambiae* (former S form) and *An. coluzzii* (former M form) in Togo [30]. This situation is evidence of the spread of *kdr* resistance within *Anopheles* populations. This study focuses on three different agricultural areas: vegetable production (Baguida), rice cultivation (Kovié) and cotton production (Kolokopé). The development and spread of the resistance in malaria vectors has been attributed to intensive use of insecticides in agriculture, particularly in cotton cultivation [31–33]. The development of illegal insecticide-selling markets in rural areas also plays a key role in the resistance [9]. Agricultural practices create numerous trenches that retain rain and water from irrigation systems. In Cameroon, *An*. *gambiae* s.l. females were reported to frequently lay their eggs in breeding sites located around agricultural settings, suggesting that larvae may undergo a selection pressure from agricultural pesticides that promotes the emergence of resistance [34]. Evidence for this assumption was recently reported in Burkina-Faso [35]. Agriculture is the main source of income for most of the population of Togo (70%) [6] and to date agricultural practices are expanding in rural areas and in city outskirts. Indeed, many studies reported that in Togo crop protection practices are primarily based on the excessive use of synthetic pesticides, which in most cases includes pyrethroids and organophosphates [36, 37]. Agboyi et al. [37] reported that farmers still use organochlorides, although they are obsolete. In cotton and vegetable fields, insecticide residues are accumulated in the soil and during rainy seasons, breeding sites are permanently contaminated; larvae are then constantly under insecticide pressure.

Pyrethroid resistance in *Anopheles* population is worrying and it can compromise vector control strategies, such as the use of pyrethroid-only LLINs. In addition to the kdr 1014S and 1014F alleles identified in this study, Djègbè et al. [38] previously reported the presence of the 1575Y allele in *An. coluzzii* and *An. gambiae* from Kovié and Nangbéto, suggesting the situation is worsening. Furthermore, recent study conducted at Kolokopé showed high levels of resistance in *An. gambiae* s.l. (Ahadji-Dabla et al. pers. commun.). The frequency of kdr-east found at Kolokopé (2012) was 0.043, which is very low but not insignificant. This mutation was found for the first time at Nangbeto in 2015 [38], a locality pertaining to the same region as Kolokopé; suggesting that kdr-east mutation appeared in *Anopheles* populations of this region before being reported in 2018. *Kdr*-*east* allele was found in West Africa first in Benin in *An. arabiensis* [39] and then in Burkina Faso in *An. coluzzii*, *An. gambiae*, and *An. arabiensis* [40]. Recently, it was reported in both *An. coluzzii* and *An. gambiae* in Togo [38]. In this study, it was identified only in *An. gambiae*. It could probably be present in other species.

Insensitive acetylcholinesterase was found in both *An. coluzzii* and *An. gambiae*, and in the hybrid form as well. The presence of this mutation in *Anopheles* population suggests resistance to organophosphates and/or carbamates, which are also used by farmers [37]. *Ace*-*1* was found in *An. coluzzii* at Kovié (2013 and 2016) and the frequencies were lower than those of Baguida (2012 and 2013). The allele was also previously reported at Lomé [41] and Kovié and Nangbéto [39]. Unfortunately, the duplication of the copy number of *ace-1* allele was reported for the first time at Kovié and was associated to carbamate resistance [42] and Baguida [43], suggesting the reinforcement and spread of this resistance. The situation is worrying because this could represent a serious threat for resistance management strategies [43].

Based on the results of this study and the previous ones, the alarm is being sounded, to draw the attention of decision-makers, especially the NMCP, to the urgent need of resistance management programme implementation.

## Conclusion

The *Anopheles gambiae* s.l. population in Togo was resistant to almost all the insecticides during the 3-year survey except to some organophosphates. Three types of resistance alleles were identified such as 1014F, 1014S and 119S. These types of resistance, coupled with those reported previously, represent a serious concern and as such a strategy should be devised to successfully control malaria vectors in Togo.

## Additional file

**Additional file 1: Table S1.** Knockdown times (KDTs) calculated after exposure of *Anopheles gambiae* s.l. to 4% DDT, 0.05% deltamethrin, and 0.75% permethrin.

## Data Availability

All data generated or analysed during this study are included in this manuscript.

## Abbreviations

ace-1: insensitive acetylcholinesterase
CTAB: cetyl trimethyl ammonium bromide
DNA: desoxyribonucleic acid
DDT: dichlorodiphenyltrichloroethane
IRSS: Institut de Recherche en Sciences de la Santé
kdr: knock down resistance
LLIN: long-lasting insecticide-treated nets
NMCP: National Malaria Control Programme
PCR: polymerase chain reaction
PNLP: Programme National de Lutte contre le Palusisme
(RR): resistant homozygous
SINE 200: Short INterspersed Elements
(SS): susceptible homozygous
WHO: World Health Organization

## References

[1] WHO (2017). World malaria report, 2017.

[2] WHO (2018). World malaria report, 2018.

[3] PNLP. Programme national de lutte contre le paludisme. Rapport annuel, 2013. Togo.

[4] PNLP. Programme National de Lutte contre le Paludisme. Rapport d’activités, 2006. Togo.

[5] PNLP. Programme National de Lutte contre le Paludisme. Rapport annuel, 2016. Togo.

[6] PNLP. Programme National de Lutte contre le Paludisme. Rapport d’activités, 2009. Togo.

[7] Amoudji AD, Ahadji-Dabla KM, Konaté L, Ketoh GK, Apétogbo YG, Glitho IA (2015). Evaluation de l’efficacité des moyens de lutte antivectorielle utilisés dans les ménages au Togo. J Rech Sci Univ Lomé (Togo), Série A.

[8] Ketoh KG, Morgah K, Akogbeto M, Faye O, Glitho IA (2005). Insecticide susceptibility status of *Anopheles* populations in Togo. J Rech Sci Univ Lomé (Togo), Série A.

[9] Ahadji-Dabla KM, Ketoh GK, Nyamador WS, Apetogbo GY, Glitho IA (2014). Susceptibility to DDT and pyrethroids, and detection of knockdown resistance mutation in *Anopheles gambiae* sensu lato in Southern Togo. Int J Biol Chem Sci..

[10] Ahadji-Dabla KM, Nyamador WS, Amoudji AD, Apétogbo YG, Oboussoumi KF, Aawi A (2015). Susceptibility of a malaria vector *Anopheles gambiae* s.l. (Diptera: Culicidae) to WHO recommended insecticides in Togo (West Africa**)**. J Entomol Zool Stud..

[11] Vulule JM, Beach RF, Atieli FK, McAllister JC, Brogdon WG, Roberts JM (1999). Elevated oxidase and esterase levels associated with permethrin tolerance in *Anopheles gambiae* from Kenyan villages using permethrin-impregnated nets. Med Vet Entomol.

[12] Hemingway J, Hawkes NJ, McCarroll L, Ranson H (2004). The molecular basis of insecticide resistance in mosquitoes. Insect Biochem Mol Biol.

[13] Martinez-Torres D, Chandre F, Williamson M, Darriet F, Bergé J, Devonshire AL (1998). Molecular characterization of pyrethroid knockdown resistance (kdr) in the major malaria vector *Anopheles gambiae* s.s. Insect Mol Biol.

[14] Ranson H, Jensen B, Vulule J, Wang X, Hemingway J, Collins F (2000). Identification of a point mutation in the voltage-gated sodium channel gene of Kenyan *Anopheles gambiae* associated with resistance to DDT and pyrethroids. Insect Mol Biol.

[15] Weill M, Malcolm C, Chandre F, Mogensen K, Berthomieu A, Marquine M (2004). The unique mutation in ace-1 giving high insecticide resistance is easily detectable in mosquito vectors. Insect Mol Biol.

[16] Padonou GG, Sezonlin M, Ossé R, Aizoun N, Oké-Agbo F, Oussou O (2012). Impact of three years of scale Indoor Residual Spraying (IRS) and Insecticide Treated Nets (ITNs) interventions on insecticide resistance in *Anopheles gambiae* s.l. in Bénin. Parasit Vectors..

[17] Aïkpon R, Sèzonlin M, Ossè R, Akogbéto M (2014). Evidence of multiple mechanisms providing carbamate and organophosphate resistance in field *Anopheles gambiae* population from Atacora in Benin. Parasit Vectors..

[18] Ngufor C, N’Guessan R, Fagbohoun J, Subramaniam K, Odjo A, Fongnikin A (2015). Insecticide resistance profile of *Anopheles gambiae* from a phase II field station in Cové, southern Benin: implications for the evaluation of novel vector control products. Malar J..

[19] Dabiré KR, Diabaté A, Namontougou M, Djogbénou L, Kengne P, Simard F (2009). Distribution of insensitive acetylcholinesterase (ace-1R) in *Anopheles gambiae* s.l. populations from Burkina Faso (West Africa). Trop Med Int Health..

[20] Dabiré KR, Diabaté A, Namountougou M, Djogbénou L, Wondji C, Chandre F, et al. Trends in insecticide resistance in natural populations of malaria vectors in Burkina Faso, West Africa: 10 years’ surveys. In: Perveen F, ed. Insecticides pest engineering. 2012. vol. 22, p. 479–502.

[21] Dabiré RK, Namountougou M, Diabaté A, Soma DD, Bado J, Hyacinthe K (2014). Distribution and Frequency of kdr Mutations within *Anopheles gambiae* s.l. Populations and First Report of the Ace.1G119S Mutation in *Anopheles arabiensis* from Burkina Faso (West Africa). PLoS One.

[22] Hunt RH, Fuseini G, Knwoles S, Stiles-Ocran J, Verster R, Kaiser ML (2011). Insecticide resistance in malaria vector mosquitoes at four localities in Ghana, West Africa. Parasit Vectors..

[23] Baffour-Awuah S, Annan AA, Maiga-Ascofare O, Soma DD, Adjei-Kusi P, Owusu-Dabo E (2016). Insecticide resistance in malaria vectors in Kumasi, Ghana. Parasit Vectors..

[24] Nwane P, Etang J, Chouaїbou M, Toto JC, Mimpfoundi R, Simard F (2011). Kdr-based insecticide resistance in *Anopheles gambiae* s.s. populations in Cameroon. BMC Research Notes..

[25] Antonio-Nkondjio C, Poupardin R, Fossog Tene B, Kopya E, Costantini C, Awono-Ambene P (2016). Investigation of mechanisms of bendiocarb resistance in *Anopheles gambiae* populations from the city of Yaoundé, Cameroon. Malar J..

[26] Wandjala CL, Kweka EJ (2018). Malaria vectors insecticides resistance in different agrosystems in Western Kenya. Front Public Health..

[27] WHO (1998). Tests procedures for insecticide resistance monitoring in malaria vectors, bioefficacy and persistence of insecticides on treated surfaces.

[28] WHO (2013). Test procedures for insecticide resistance monitoring in malaria vectors mosquitoes.

[29] Santolamazza F, Mancini E, Simard F, Qi Y, Tu Z, Torre A (2008). Insertion polymorphisms of SINE200 retrotransposons within speciation islands of *Anopheles gambiae* molecular forms. Malar J..

[30] Ketoh GK, Ahadji-Dabla KM, Dery D, Chabi J, Glitho IA, Brengues C, et al. Seasonal distribution and insecticide resistance in *Anopheles gambiae* s.l. in Lomé. Poster. 2009, 5th MIM Conference.

[31] Elissa N, Mouchet J, Riviere F, Meunier JY, Yao K (1993). Resistance of *Anopheles gambiae* s.s. to pyrethroids in Côte d’Ivoire. Ann Soc Belg Med Trop.

[32] Diabate A, Baldet T, Chandre F, Akogbeto M, Guiguemde TR, Darriet F (2002). The role of agricultural use of insecticides in resistance to pyrethroids in *Anopheles gambiae* s.l. in Burkina Faso. Am J Trop Med Hyg.

[33] Yadouleton A, N’Guessan R, Allagbé H, Asidi A, Boko M, Ossè R (2010). The impact of the expansion of urban vegetable farming on malaria transmission in major cities of Benin. Parasit Vectors..

[34] Antonio-Nkondjio C, Fossog BT, Ndo C, Djantio BM, Togouet SZ, Awono-Ambene P (2011). *Anopheles gambiae* distribution and insecticide resistance in the cities of Douala and Yaounde (Cameroon): influence of urban agriculture and pollution. Malar J..

[35] Hien AS, Soma DD, Hema O, Bayili B, Namountougou M, Gnankine O (2017). Evidence that agricultural use of pesticides selects pyrethroid resistance within *Anopheles gambiae* s.l. populations from cotton growing areas in Burkina Faso, West Africa. PLoS One.

[36] Agboyi LK, Djade KM, Ahadji-Dabla KM, Ketoh GK, Nuto Y, Glitho IA (2015). Vegetable production in Togo and potential impact of pesticide use practices on the environment. Int J Biol Chem Sci..

[37] Mondedji AD, Nyamador WS, Amevoin K, Adéoti R, Abbey GA, Ketoh GK (2015). Analyse de quelques aspects du système de production légumière et perception des producteurs de l’utilisation d’extraits botaniques dans la gestion des insectes ravageurs des cultures maraîchères au Sud du Togo. Int J Biol Chem Sci..

[38] Djègbè I, Akoton R, Tchigossou G, Ahadji-Dabla KM, Atoyebi SM, Adéoti R (2018). First report of the presence of L1014S Knockdown-resistance mutation in *Anopheles gambiae ss* and *Anopheles coluzzii* from Togo, West Africa. Welcome Open Research..

[39] Djègbè I, Boussari O, Sidick A, Martin T, Ranson H, Chandre F (2011). Dynamics of insecticide resistance in malaria vectors in Benin: first evidence of the presence of L1014S kdr mutation in *Anopheles gambiae* from West Africa. Malar J..

[40] Namountougou M, Diabate A, Etang J, Bass C, Sawadogo SP, Gnankiné O (2013). First report of the L1014S *kdr* mutation in wild populations of *Anopheles gambiae* M and S molecular forms in Burkina Faso (West Africa). Acta Trop.

[41] Ahadji-Dabla KM, Amoudji AD, Dery DB, Apétogbo YG, Ketoh GK, Glitho IA (2017). Susceptibility to carbamate and organophosphate, and ace-1 allele in *Anopheles gambiae* s.l. from pyrethroid resistance areas in the city of Lomé, Togo, West Africa. Int J Biol Med Res..

[42] Edi CV, Djogbenou L, Jenkins AM, Regna K, Muskavitch MA, Poupardin R (2014). CYP6 P450 enzymes and ACE-1 duplication produce extreme and multiple insecticide resistance in the malaria mosquito *Anopheles gambiae*. PLoS Genet.

[43] Assogba BS, Milesi P, Djogbenou LS, Berthomieu A, Makoundou P, Baba-Moussa LS (2016). The *ace*-*1* locus is amplified in all resistant *Anopheles gambiae* Mosquitoes: fitness consequences of homogeneous and heterogeneous duplications. PLoS Biol.

