# Supplementary material for: Insecticide resistance profiles of Anopheles gambiae s.l. in Togo and genetic mechanisms involved, during 3-year survey: is there any need for resistance management?
Source: Malar J. 2019 May 22;18:177. doi: 10.1186/s12936-019-2813-z (PMC6530008; doi:10.1186/s12936-019-2813-z)
Supplement: Supplementary file 1 — Additional file 1: Table S1. Knockdown times (KDTs) calculated after exposure of Anopheles gambiae s.l. to 4% DDT, 0.05% deltamethrin, and 0.75% permethrin. [file 12936_2019_2813_MOESM1_ESM.docx]

**Additional file 1**

**Table S1** Knockdown times (KDTs) calculated after exposure of *An. gambiae s.l.* to 4% DDT, 0.05% deltamethrin, and 0.75% permethrin.

| **Year** | **Locality** | **4% DDT** | | | **0.05% deltamethrin** | | | **0.75% permethrin** | | |
| --- | --- | --- | --- | --- | --- | --- | --- | --- | --- | --- |
|  |  | **N** | **KDT_50_**  **[95%CI] min** | **KDT_95_**  **[95%CI] min** | **N** | **KDT_50_**  **[95%CI] min** | **KDT_95_**  **[95%CI] min** | **N** | **KDT_50_ [95%CI] min** | **KDT_95_**  **[95%CI] min** |
| **2012** | Baguida | 103 | a | a | 99 | 42.56  [38.18-48.28] | 131.73  [101.36-198.77] | c | c | c |
|  | Kolokopé | 95 | a | a | 101 | 94.06  [70.81-160.98] | 464.73  [239.06-1731.3] | c | c | c |
|  | Kovié | 98 | a | a | 95 | 44.89  [41.22-49.52] | 143.5  [114.82-197.04] | c | c | c |
| **2013** | Baguida | 97 | a | a | 87 | 24.71  [22.4-27.12] | 90.78  [76.35-113.49] | 89 | b | b |
|  | Kolokopé | 96 | a | a | 74 | 73.45  [59.69-164.31] | 136.56  [88.52-797.9] | 80 | b | b |
|  | Kovié | 90 | a | a | 90 | 42.66  [39.26-46.82] | 131.1  [106.29-176.39] | 90 | b | b |

**Table S1** (*Cont.*)

| **Year** | **Locality** | **4% DDT** | | | **0.05% deltamethrin** | | | **0.75% permethrin** | | |
| --- | --- | --- | --- | --- | --- | --- | --- | --- | --- | --- |
|  |  | **N** | **KDT_50_**  **[95%CI] min** | **KDT_95_**  **[95%CI] min** | **N** | **KDT_50_**  **[95%CI] min** | **KDT_95_**  **[95%CI] min** | **N** | **KDT_50_ [95%CI] min** | **KDT_95_**  **[95%CI] min** |
| **2016** | Baguida | 121 | a | a | 123 | 56.92  [51.55-64.68] | 203.99  [154.86-304.06] | 72 | 193.46  [108.14-1745.58] | 1001.1  [307.6-97251] |
|  | Kolokopé | 69 | a | a | 67 | 127.76  [87.9-330.4] | 586.03  [254.57-5310] | 93 | a | a |
|  | Kovié | 98 | a | a | 91 | 168.25  [106.8-539.6] | 898.02  [342.8-11333] | 73 | 396.28  [155.34-20666] | 4956.2  [747-7289] |

N: number of individuals, KDT_50_: Knockdown time for 50% individuals, KDT_95_: Knockdown time for 95% individuals, a = No knockdown effect, b = Individuals knocked down well below 50% within the 60 minutes of exposure, c = Insecticide not tested.
